# Supporting contraceptive self-care and reproductive empowerment with a digital health game in Barbados: Development and Pre-implementation study for What’s My Method?

**DOI:** 10.12688/gatesopenres.15376.2

**Published:** 2024-11-21

**Authors:** Elena Bertozzi, Clara Bertozzi-Villa, Erin Sabato, Nicole Alleyne, Sonia Watson-Miller, Tiffany Jordan, Anderson Langdon

**Affiliations:** 1 Game Design & Development, Quinnipiac University, Hamden, Connecticut, 06518, USA; 2 Division of Maternal Fetal Medicine, Department of Obstetrics & Gynecology and Women’s Health, Montefiore Health System, Bronx, New York, USA; 3 Office of Global Engagement, Quinnipiac University, Hamden, Connecticut, 06518, USA; 4 Hibiscus Health Caribbean, Bridgetown, Saint Michaels, Barbados; 5 Nursing Department, Barbados Community College, Saint Michael, Barbados; 6 Barbados Family Planning Association, Bridgetown, Saint Michaels, Barbados

**Keywords:** contraceptive self-efficacy, videogame, reproductive empowerment, family planning method, education

## Abstract

Effective contraceptive education is essential to reducing unwanted pregnancy, increasing uptake of modern contraceptive methods, and thoughtfully planning desired births. New World Health Organization (WHO) and family planning organization guidelines recommend situating contraceptive education and counseling within a broader context of self-care that emphasizes individual agency and reproductive empowerment. Digital health interventions, and games for health specifically, have been validated as effective and scalable tools for self-guided and interactive health education, especially among younger tech-savvy individuals. Barbados currently supplements provider-based contraceptive counseling with analog materials (pamphlets and posters) and informational videos that play on a screen in the waiting room. As part of an implementation framework, this study seeks to conduct a formative evaluation of the What’s My Method? (WMM) game intervention as a tool to support contraceptive counseling and increase reproductive empowerment among childbearing persons in Barbados. We test-deployed the WMM game in Bridgetown, Barbados, conducting playtests and unstructured discussions with prototypes of the WMM game among three groups of stakeholders (youth contraception ambassadors: n=8; healthcare providers: n=7; and nursing students: n=27) to determine acceptability of the intervention, efficacy of the game as a learning tool, and willingness to adopt the tool in their healthcare context. Feedback on acceptability of the game was largely positive. Detailed constructive comments informed modifications and improvements to the game. The questionnaire used to assess contraceptive knowledge gain did not prove effective. Results indicate that the WMM game is well-received and accepted by the healthcare professionals who would be deploying it. This pilot testing has informed the design of the modified WMM for a randomized controlled trial (RCT) to test the deployment of the game in a healthcare setting.

## Introduction

### Background

Contraceptive self-care has been identified by the WHO as critical to achieving milestones for female empowerment and well-being ( World Health Organization, 2022). This focus reflects a shift in global public health attitudes regarding contraceptive education from a top-down approach—where childbearing persons receive directives, often from healthcare professionals and/or government institutions—towards an approach that encourages self-determination and agency ( Hamidi *et al.*, 2018). Reproductive empowerment is a means of implementing and interpreting contraceptive self-care and has been defined as:

Both a transformative process and an outcome, whereby individuals expand their capacity to make informed decisions about their reproductive lives, amplify their ability to participate meaningfully in public and private discussions related to sexuality, reproductive health and fertility, and act on their preferences to achieve desired reproductive outcomes, free from violence, retribution, or fear. ( Edmeades *et al.*, 2018)

Improving pregnancy planning and preventing unintended pregnancy is a WHO priority, one of the US Healthy People Objectives ( Office of Disease Prevention and Health Promotion, 2023), and a public health goal of Barbados. Implementing contraceptive counseling and education through the lens of reproductive empowerment requires that the focus of intervention be on the childbearing persons and their needs. Ideally, in addition to describing associated risks, this counseling also reviews the many non-contraceptive benefits of hormonal birth control methods, including menstrual regulation and relief of symptoms associated with conditions such as endometriosis and polycystic ovarian syndrome (PCOS) ( Kopp Kallner, 2018). The selection of a contraceptive method should be made with an understanding of the potential costs and benefits of each method. Some studies have been done to measure the impact of contraceptive self-efficacy on increased adoption of contraception. However, most studies focused solely on condom use ( Burke *et al.*, 2021; Whiting-Collins *et al.*, 2020). These studies show that higher rates of self-efficacy relative to condoms result in increased condom use and recommend additional research measuring efficacy with other forms of contraception ( Burke *et al.*, 2021).

There is clear need for improvement in contraceptive counseling in Barbados. As per the United Nations dashboard, the contraceptive prevalence rate for any method among women aged 15–49 was 50% in 2022 ( United Nations, 2023). Providers report that abortion is often used as birth control, and the fertility rate is declining (1.6 live births per woman in 2023). Childbearing women may also receive advice propagating unscientific concerns about side effects which makes them reluctant to adopt modern methods. Low uptake of modern contraceptive methods leads to outcomes including unwanted pregnancy and use of abortion as birth control ( Bearak *et al.*, 2022; Claridge, 2021; Gipson *et al.*, 2008).

Barbados includes contraceptive counseling (CC) as a standard part of post-natal and pre-abortion care. CC is an essential aspect of healthcare for women and child-bearing individuals. This counseling should be thorough, unbiased, and tailored to each patient’s specific needs and health conditions. Providing CC is complicated by patient use of the internet which provides a plethora of information that can be difficult to filter and interpret as well as abundant misinformation which must be countered. The proliferation of misinformation is accelerated by emerging technology use such as influencers on TikTok and YouTube ( Pfender & Devlin, 2023). Barbadian clinics have not integrated interactive digital technology to support CC; information about methods is delivered using videos, pamphlets and posters.

Digital health interventions have been identified as successful high-impact practices to support healthy reproductive behaviors ( Dehlendorf *et al.*, 2019; Stephenson *et al.*, 2020). Digital health interventions supporting contraceptive self-efficacy include SMS campaigns ( Chukwu *et al.*, 2021; Laidlaw *et al.*, 2017), artificial intelligence-based chatbots ( Wang *et al.*, 2022), and interactive websites ( Lepore *et al.*, 2024) which provide information and offer tools to help choose appropriate methods. These interventions demonstrate that digital media are an effective way to reach the target audience and communicate information about reproductive health.

Demonstrating the impact of interventions on reproductive empowerment is complicated. There are many different forms of contraception, which vary among mechanism of action, route of administration, frequency of dosing, side effects, and many other factors. Understanding and remembering these nuances is difficult even for healthcare providers. Videogames can be useful for this type of learning. Games are a validated means of presenting complex information in a format that provides context-based learning, encourages engagement through fun, and rewards success ( Squire, 2011). People are more likely to seek out information and remember it if it is contextualized and relevant ( Bado, 2022). Games focused on sexual health and education have been successfully deployed in other settings ( Bertozzi *et al.*, 2018; Fiellin *et al.*, 2017; Haruna *et al.*, 2018).

This project is a development and pre-implementation study aimed at gathering information from stakeholders to determine if the WMM game would be an accepted and effective tool to support CC in their practice. Game design is an iterative process that collects feedback at several stages during the design process. Nilsen *et al.*, 2020 During this study, a beta version of the game in two parts was loaded on tablets and provided to three groups of stakeholders. There are three sections in the game: Reproductive Anatomy, How Methods Work, and Helping Couples. These sections are described in detail below.

### Aims

The aims of this research were to: 1) Introduce the beta WMM game to current and future healthcare providers as a tool to support educating couples about contraception through the lens of reproductive empowerment. 2) Observe users while they test the game to determine any usability issues or technical difficulties. 3) Assess the utility and accuracy of the pre/post questionnaire. 4) Conduct unstructured interviews with key stakeholders (healthcare providers at clinics, faculty at nursing school) to understand their needs and desired outcomes.

### Theoretical frameworks

Interactive learning games leverage several theoretical models for achieving desired behavior change. These include social learning theory and social cognitive theory, both of which posit that there are interrelated factors (embedded learning, repetition, enjoyment) that influence behavior after the completion of the experience ( Krath *et al.*, 2021). The efficacy of games for health and app-based gamification of health-focused education is now well established ( Haoran *et al.*, 2019; Sharifzadeh *et al.*, 2020). The WMM game is designed to employ known educational learning game principles, utilize research-standard tools ( Duncan *et al.*, 2014), and include learning through empathy. Empathy is increasingly recognized as an important component of healthcare education and science learning ( Mikkonen *et al.*, 2015; Zeyer & Dillon, 2019).

Conversations about sexual reproduction, contraception, and family planning are often complicated by shame, cultural expectations, and countless other factors. Providing information in a format that allows participants privacy and freedom to explore topics without judgement can enable more constructive conversations ( Bertozzi *et al.*, 2018). The WMM game evokes empathetic behavior by asking players to help avatar couples in the game and select a method that best meets the couples’ physiological and behavioral needs. To succeed, players must literally listen to the expressed needs of the people in the game and observe their interactions with their partners. The in-game conversations reinforce information about how methods work, how they fail, and how side effects can be both negative and positive in different circumstances. Players are motivated by compelling narratives to find the information needed to pick an optimal method that will satisfy both partners.

Measuring contraceptive knowledge is complicated by many factors including health literacy, assumptions about what constitutes basic contraceptive education, and participant willingness to complete a quiz. It is difficult to word text-based questions to accurately determine respondent knowledge. Our pre and post test questions were based on simplified questions from the Contraceptive Knowledge Assessment (CKA) ( Haynes *et al.*, 2017). We sought to determine if this metric would be useful for our purposes or if we should focus on collecting performance metrics from player behavior.

In order for novel digital health interventions to become widely adopted, it is necessary for them to be integrated into routine healthcare practice. Thus, assessing the efficacy of the intervention should include the perspective of stakeholders including healthcare providers, educators, and policymakers. Implementation science research frameworks provide models for organizing research inclusive of intervention efficacy and adoption by practitioners ( Damschroder *et al.*, 2022; Proctor *et al.*, 2011), Our process was informed by the Exploration, Preparation, Implementation, Sustainment (EPIS) framework to ensure consideration of the complex interactions between the game intervention (WMM), the intended audience, and healthcare providers ( Nilsen *et al.*, 2020). This study focuses on the first phase of the EPIS model: exploration, in which stakeholders consider the health needs of their patient populations and identify the best intervention to address those needs ( Aarons *et al.*, 2011)

## Videogame intervention: The What’s My Method? game

### Development

The first version of the intervention was as an addendum to the **My Future Family** (MFF) game funded by Grand Challenges in Global Health ^1^ in 2016 and deployed ^2^ in Mysore and Chennai, India in 2017 and 2018 ( Bertozzi *et al.*, 2018; Bertozzi *et al.*, 2021). The MFF game was aimed at school-age children who otherwise received no, or very little, education about sex. It provided information about sexual and reproductive anatomy, and collected data from gameplay about family planning intentions. Post deployment qualitative interviews in India recommended adding information about contraceptive methods and modalities ( Bertozzi *et al.*, 2021). The WMM addendum was designed to deliver information about contraception by providing patients with foundational knowledge about methods, and then engaging their new knowledge to assist in-game couples with method selection. Each couple has different lifestyles, physiological needs, and personal preferences. Players have to research methodologies and side effects of methods to find the one(s) best suited to each couple.

Due to the COVID pandemic, the WMM addendum was never deployed or tested in the field, though we were able to conduct informal usability testing on reproductive-age volunteers. In 2023, we began a partnership with the Barbados Family Planning Association and received funding to update WMM as a standalone game focused on supporting contraceptive counseling. The original version of WMM had three sections: Reproductive Anatomy, Contraceptive Methods and Modalities, and Couples. One of the goals of this pre-implementation study was to test and design updates for each of these sections, including integration of lessons learned from past deployments and addressing the needs of the new target audience informed by the EPIS framework ( Nilsen *et al.*, 2020). The exercise of identifying user needs is integrated into standard game development which is an iterative process requiring testing on the target audience during the production cycle to ensure that the game is fun, easy to use and understand, and achieves the game goals. We scheduled the pre-implementation study after building a prototype of the new graphic design for the game but before the design had been extended to the Couples section.

### Reproductive anatomy

The focus groups in India for the My Future Family game ( Bertozzi *et al.*, 2018) revealed that players could not understand how contraception works without understanding sexual reproductive anatomy. Thus, the MFF game includes a section where players identify reproductive body parts and their functions. An unanticipated positive result of this part of the game was that it normalized study participant verbalization of anatomic vocabulary during gameplay and in post play conversations (i.e. many students were very uncomfortable saying “vagina” before playing the game, and afterwards did so without hesitation). We attempted a formal pre/post assessment of knowledge gain in the MFF deployment using a drag and drop quiz, but this was not well-received by players and many declined to complete the post-game assessment ( Bertozzi *et al.*, 2021). Lessons learned from the India deployments guided our decision to both retain a reproductive anatomy section in the new WMM game and to use this segment as a knowledge assessment tool. This section assesses players knowledge of the names and functions of the body parts by seeing how quickly they solve the puzzle and how often they make mistakes. In the RCT we will see if familiarity with reproductive anatomy facilitates frank conversations with healthcare providers as part of our assessment of reproductive empowerment.

**Figure 1.**
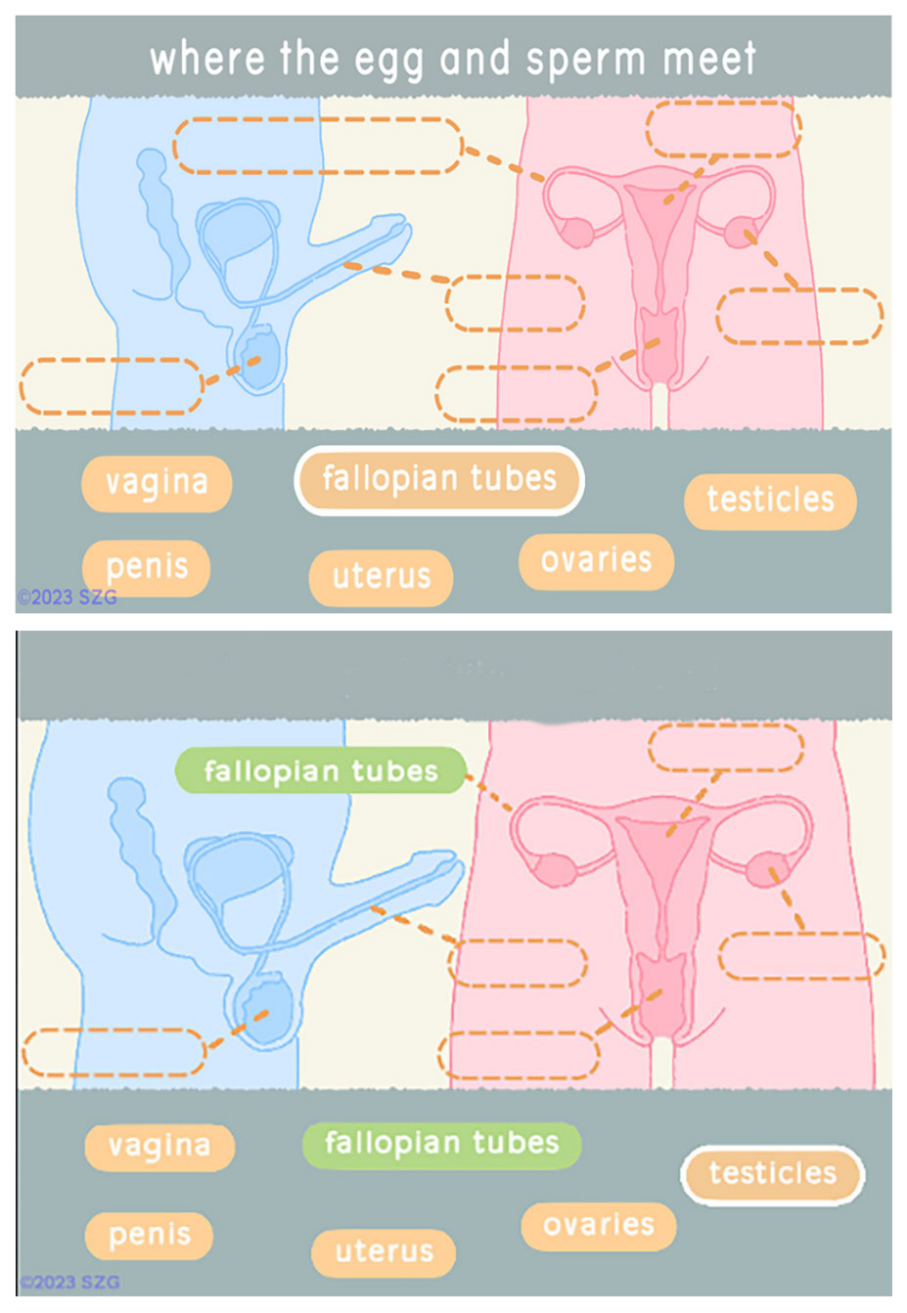
Screenshots from revised reproductive anatomy section deployed in Barbados. Reproduced with permission from SolitonZ Games.

Players in Barbados tested the updated Reproductive Anatomy section ( Figure 1). When players click on a term, it is highlighted and a question appears. They then select a dashed oval. If the selection is correct, the text fills the oval which becomes green. If the answer is incorrect the dashed oval flashes red and the text is not placed there. Players cannot proceed until each oval is filled correctly.

### Contraceptive methods and modalities

**Figure 2.**
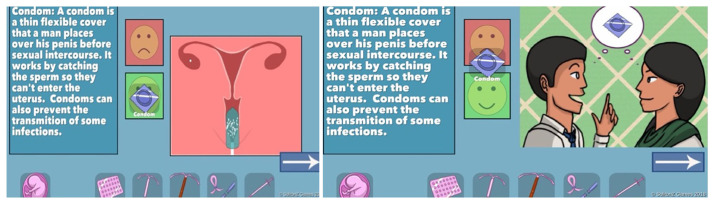
Screenshots of condom information from original WMM methods section. Reproduced with permission from SolitonZ Games.

The first version of the WMM game includes seven methods: Condom, Pill, Copper IUD, Hormone IUD, Tubal Ligation/Vasectomy, Depo-Provera Injection, and None ( Figure 2). Players can see a short animation for each method that explains how it works and how it might fail. For example, a condom works by physically covering the penis and blocking sperm from entering the vagina and therefore reaching the egg. It does not work if you forget to buy one or use it incorrectly. The animations engage players by depicting cartoon facial expressions of emotions such as concern, embarrassment, and delight. This section of the game was very well received by test users in the India deployment ( Bertozzi *et al.*, 2021). However, healthcare provider reviewers requested that the game include significantly more information, including additional methods, discussion of side effects (both positive and negative), general efficacy (how often the method fails), duration of the method, and timeline for return to fertility.

**Figure 3.**
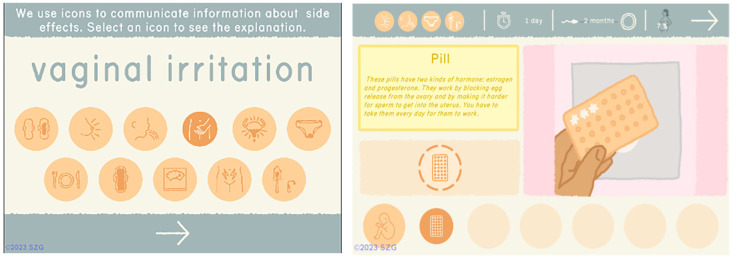
New icons and Methods section tested in Barbados. Reproduced with permission from SolitonZ Games.

One of the goals of our games is to provide complex, scientifically accurate information in formats that can be understood across a wide range of literacy levels. The addition of so much more information about each method required the design of a series of icons to represent side effects of different methods as well as icons that could visually convey metrics such as Return to Fertility, Failure Rate, and Duration. In Barbados, participants tested the legibility of the new icons and the revised Contraceptive Methods section, which at that point included 12 methods ( Figure 3).

### Couples

**Figure 4.**
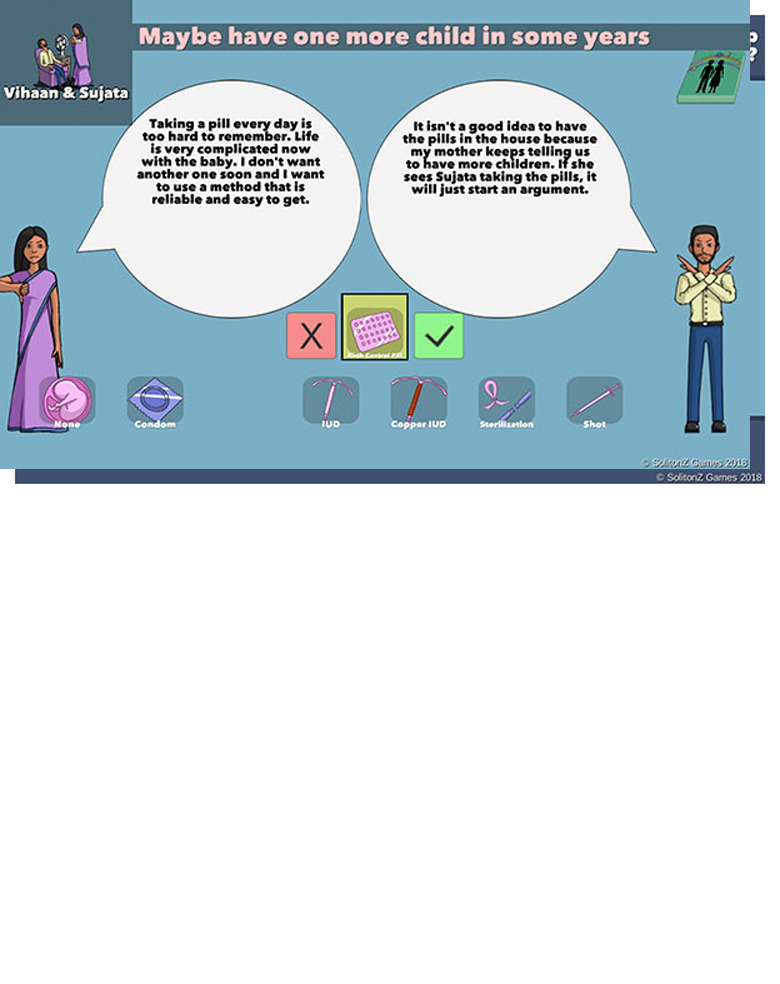
Screenshots from original WMM game deployed in Barbados. Reproduced with permission from SolitonZ Games.

We deployed the original version of the Couples section of the game ( Figure 4) to test the game mechanic and engagement among the Barbadian target audience and explained that the interface would be updated with the new graphic design and that Indian avatars and narratives would be replaced with Barbadian ones for the RCT. Players can view four couples, each with different needs and characteristics which are communicated both in on-screen text and voiceovers. Players select a method at the bottom of the screen and the couples respond to that selection in a way that explains its features. For example, Reyansh is concerned about being able to remember to take birth control pills.

## Methods

### Design

The design of the pre-implementation study sought to ensure that the research, game design, and implementation teams had input from all stakeholders. We conducted playtesting sessions and then had informal conversations with participants after they completed the post-tests. Playtesters were observed during the playtesting process to determine if there were any issues with accessing, using, or understanding the technology as this is an important part of iterative game testing. We collected qualitative and quantitative data in our pre and post-test questionnaires.

### Participants

As this was a development and pre-implementation study we used a combination of convenience and purposive sampling. The study population included volunteers from our target demographics including: staff and administration from the Barbados Family Planning Association (BFPA), faculty and students from the Nursing program at Barbados Community College (BCC), and volunteers from the Youth Advocacy Movement (YAM) who do community outreach about family planning. Many of the healthcare providers providing feedback on the utility of the tool to support contraceptive counseling are also themselves childbearing persons who are the intended audience of the intervention. ( Table 1). The BFPA is a hybrid governmental and non-profit organization. It was incorporated in 1967 by an Act of Parliament and provides sexual and reproductive health information, clinical services, education, training, community outreach, and research. A recruitment call was sent out to the YAM volunteers who meet regularly at the BFPA. Nursing students from BCC were recruited as play-testers who would serve both as representatives of our target population (childbearing persons in Barbados) and as future healthcare providers. BCC provides education and training for entry-level nurses and post-registration nursing programs in Barbados and attracts students from across the Caribbean. There is a renewed focus on supporting research among nursing faculty that provides experiential education opportunities for students. BCC faculty and students will be involved in the RCT.

**Table 1.** Participants.

Category	Participants (N=42), n (%)	Demographics
YAM ambassadors	8 (22)	4 female, 4 male ages (20–25)
BFPA staff and affiliates	7 (18)	6 female, 1 male ages (32–53)
BCC nursing students	27(70)	18 female, 9 male ages (20–35)

### Procedures

Playtesting and discussions with each category of participant were conducted on separate days during October 2023. The research team had 6 tablets loaded with 2 files. One was a beta version of the new WMM game with a tutorial for understanding the side effect icons, new icons to represent birth control methods, the Reproductive Anatomy puzzle, and a test version of the Methods section. The other was the India addendum with untested versions of the Methods and Couples sections.

**Figure 5.**
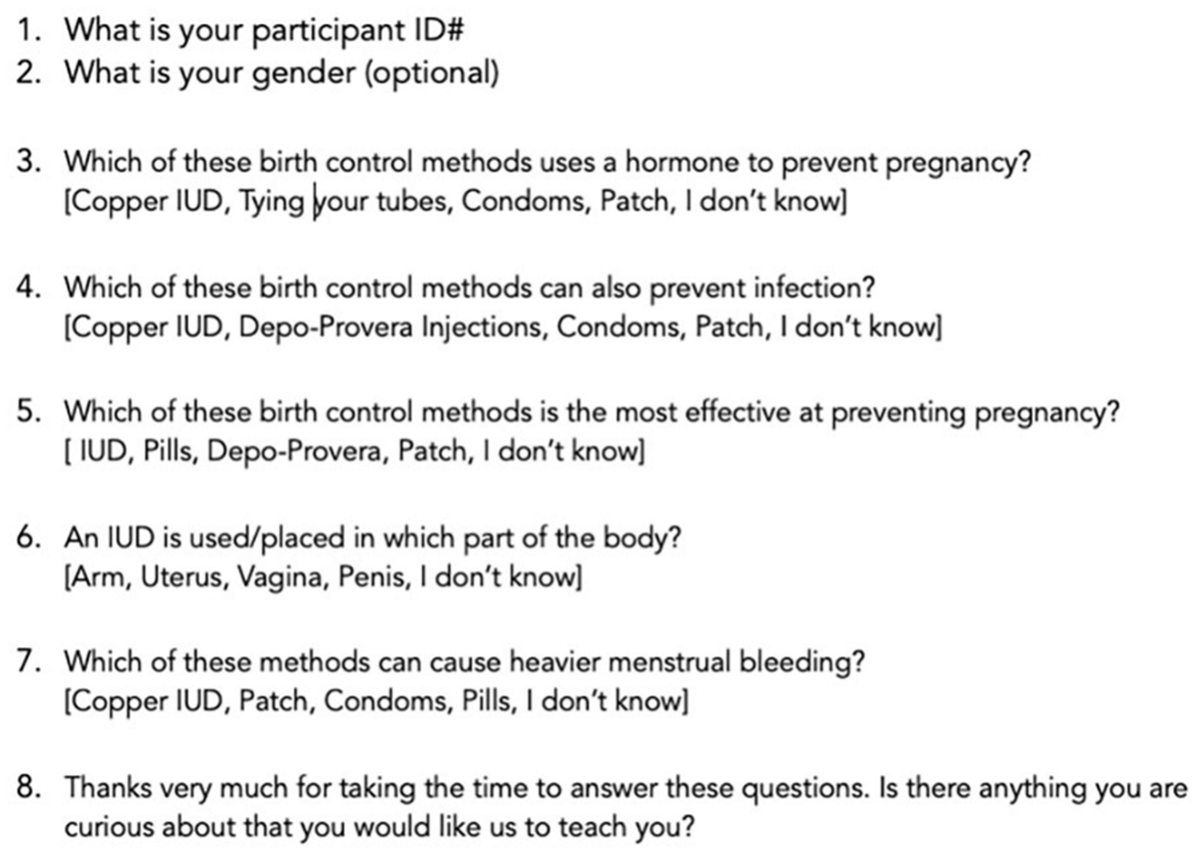
Participant knowledge pre-test.

**Figure 6.**
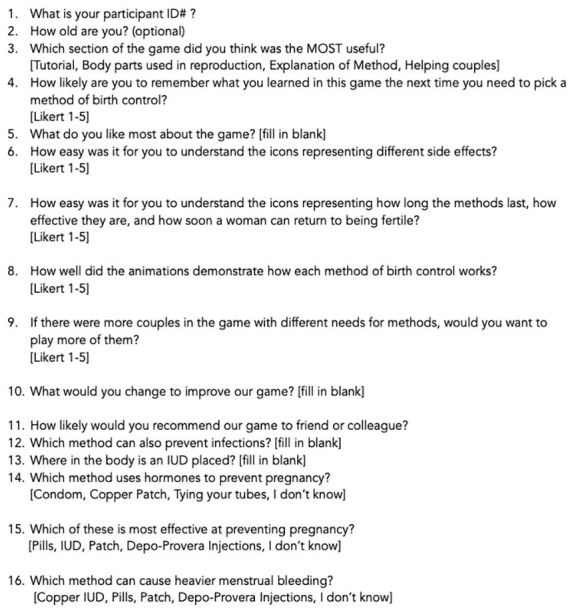
Participant knowledge post-test.

The protocol began by having participants complete paper consent forms. They then were provided with a QR code to access the pre-test ( Figure 5) on their phones. The pre-test asks for optional gender identification and 5 questions about contraceptive knowledge. Following submission of the pre-test, tablets were handed to participants. They were encouraged to work together if desired (which was necessary when participants outnumbered tablets). The following instructions were provided: Play the new version of the WMM game which includes a tutorial section with icons, the Reproductive Anatomy puzzle, and a Methods section; and then play the Couples game from the older version. They were told they could stop playing at any time and could ask questions or receive help if they were confused about how to proceed. When they were finished playing, they were provided with a different QR code for the post-test ( Figure 6) to be completed on their phones. The post-test consists of 10 questions that collect player and usability feedback on the play experience and then 5 questions about contraceptive knowledge that mirror those in the pretest, though the wording is different. Data were collected using anonymized Outlook forms stored on secure academic servers.

The YAM ambassadors who agreed to participate met with researchers at BFPA main clinic. They were all given the consent forms, then pre-test, tablets, and post-test. Some participants chose to play next to each other and discuss the process. Others sat apart and completed it on their own. Following the experience, researchers led an informal discussion where more feedback and suggestions were collected.

The BFPA staff and affiliates who participated met individually with researchers. They went through the same protocol and then provided additional verbal feedback about playing the game as a participant and how they would use the game as part of their provision of healthcare to childbearing patients and their partners.

BCC students participated in a classroom setting. Participants clustered in groups of 3 or 4 per tablet and played communally after having completed the pre-test individually on phones. Researchers noted any issues that arose during playtesting as well as main themes from the unstructured discussions following the playtests.

### Analysis

Researchers observed and documented participant behavior during playtesting sessions and post play group discussion. Salient and recurring themes were collected, and technical and usability issues were noted.

40 participants completed the pre-test, and 33 completed the post-test. Average time needed for all three steps was 47 minutes. Given that all of the participants in the pre-implementation study are either healthcare professionals or undergoing related training, we expected high rates of knowledge of reproductive anatomy and contraceptive knowledge. We sought to determine the usability of the Reproductive Anatomy puzzle and comprehensibility of the questionnaire items.

The pre-test consisted of 5 multiple choice questions about contraceptive knowledge. These questions were coded as either correct or incorrect. The post-test asked the same 5 questions in a different order with slightly different wording and were coded as correct or incorrect. Feedback on the overall playability and acceptability of the game and on specific parts of the game was collected using a 1–5 Likert scale. Two participants did not complete the pre-test and 7 did not complete the post-test. Thus, our comparison of pre/post results utilized a data set of 33 responses.

### Ethical considerations

All the study procedures were approved by the Quinnipiac Institutional Review Board (protocol #15923, approved 10/17/2023). Given the sensitive nature of the topics covered by the game, participants were informed in advance that they would see representations of reproductive processes and reproductive anatomy. They were also informed both in the consent document and verbally that they could stop participating at any time without penalty. All participants completed a written informed consent form prior to participating in the study and were verbally advised that they could stop at any time.

## Results

A two-tailed comparison of mean pre and post scores on the five contraceptive knowledge questions did not show significant improvement after having played WMM ( Table 2). One of the questions (#5) had more variations in the means. Given that the possible answers are binary (correct/incorrect) we conducted a McNemar’s test on the 33 individual responses which demonstrated slightly more impact from playing WMM, but still not significant. ( Table 3).

**Table 2.** Analysis of scores on pre/post contraceptive knowledge. t-Test: Paired Two Sample for Means comparing all Pre / Post Test Scores.

*Score range (1–5)*	*Pre-Test * *Mean Score*	*Post-Test Mean Score*
Mean	3.18	3.45
Variance	1.40	1.26
Observations	33	33
Calculated t-value:	1.11	
t Critical two-tail	±2.037	
p-value	0.246	

**Table 3.** Analysis of responses to Question #5.

McNemar's test to compare individual scores on Answer 5.	
Wrong answer Pre ->correct on Post	12
Correct on Pre to incorrect on Post	4
No change	17
Chi Square	3.0625
Alpha	0.05
Critical Value	3.84145882
p value	0.08011831

The results of the contraceptive knowledge questionnaire were of limited use in assessing contraceptive knowledge. Some questions were universally answered correctly in both pre- and post- tests, while others appeared to demonstrate confusing patterns of information acquisition. The question regarding which method also prevents STIs was universally answered correctly in both pre- and post- tests. However, participants tended to confuse the copper and hormonal IUDs. While 27.5% incorrectly indicated that copper IUDs emit hormones in the pre-test, this increased to 45% in the post-test even though participants correctly assigned the copper IUD to an avatar who refused hormone-based methods in the Couples game. There was also wide variation in response to the question assessing contraceptive efficacy, as participants felt it was unclear which parameters (usability, compliance, etc.) should be given priority when choosing an answer. The one question that demonstrated the greatest difference in pre and post responses was: “Which method can cause heavier menstrual bleeding?” however this difference was not significant ( Table 3).

The feedback on game elements demonstrated high acceptance of the game as a learning tool and positive impact on reproductive empowerment. Respondents reported appreciation of learning through interactivity, clarity of the animations and audio that demonstrated how each method works, and enjoyment of the humor and narratives in the game. On a Likert scale of 1–5: the response to “How likely are you to remember what you learned in this game the next time you need to pick a method of birth control?” was 4.18 and “How well did the animations demonstrate how each method of birth control works?” was 4.48 ( Table 4).

**Table 4.** Responses to qualitative questions on post-test.

Qualitative Feedback from post test	
Which section of the game did you think was the MOST useful?	Body Parts: 1 Methods: 12 Couples: 17 Tutorial: 3
How likely are you to remember what you learned in this game the next time you need to pick a method of birth control?	Mean 4.18 [3-5] *
How easy was it for you to understand the icons representing different side effects?	Mean 4.18 [3-5] *
How easy was it for you to understand the icons representing how long the methods last, how effective they are, and how soon a woman can return to being fertile?	Mean 4.03 [3-5] *
How well did the animations demonstrate how each method of birth control works?	Mean 4.8 [3-5] *
If there were more couples in the game with different needs for methods, would you want to play more of them?	Yes: 33 No: 1
How likely would you recommend our product to friend or colleague?	Mean 4.5 [3-5] *

*Likert scale 1–5 with range of responses in brackets.

Almost all respondents (32 of 33) said that they would have played the game for a longer period if there were more couple scenarios in the game for them to solve. This supported our observational findings during testing. As both individuals and groups played the game, they were told several times that they could stop whenever they wanted to. In almost every case, participants wanted to keep playing until they found the optimal method for each of the four couples in the game.

Suggestions for improvement also matched observational findings. The majority of players were able to easily intuit how to play the different parts of the game based on familiarity with other phone games. However, some needed assistance and indicated that the game should have a better tutorial explaining the game mechanics. Participants recommended improving the legibility of some of the icons related to side effects, methods, return to fertility, and failure rate. There were also recommendations to make the avatars, narratives, and voiceovers representative of Barbadians.

Several male respondents indicated that the couples’ narratives should be more inclusive of male perspectives and that solutions should also include the male partner. For example, the male could offer to drive his partner to medical appointments if she was otherwise likely to miss them.

## Discussion

This pre-implementation study provided both the research and game design teams with information critical to improvements to the WMM game and to the study design for the RCT.

### WMM

Although the prototype included a short tutorial demonstrating how to tap on an icon to see what it represented, some participants wanted more instruction. The WMM will therefore have a more complete tutorial depicting a cartoon hand completing the necessary actions.

**Figure 7.**
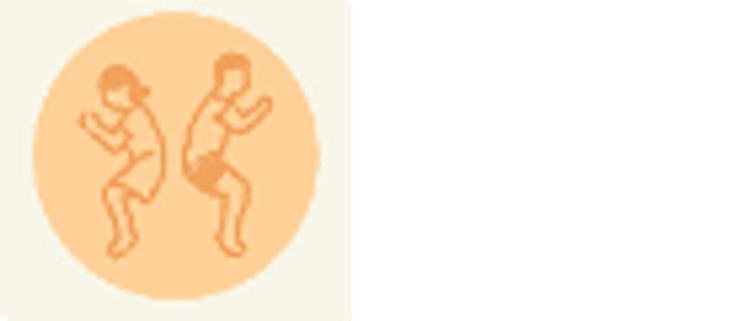
Previous and current icons for decreased libido based on user recommendations. Reproduced with permission from SolitonZ Games.

Participants were unable to understand the icon we developed for decreased libido and suggested a new one which we implemented ( Figure 7).

**Figure 8.**
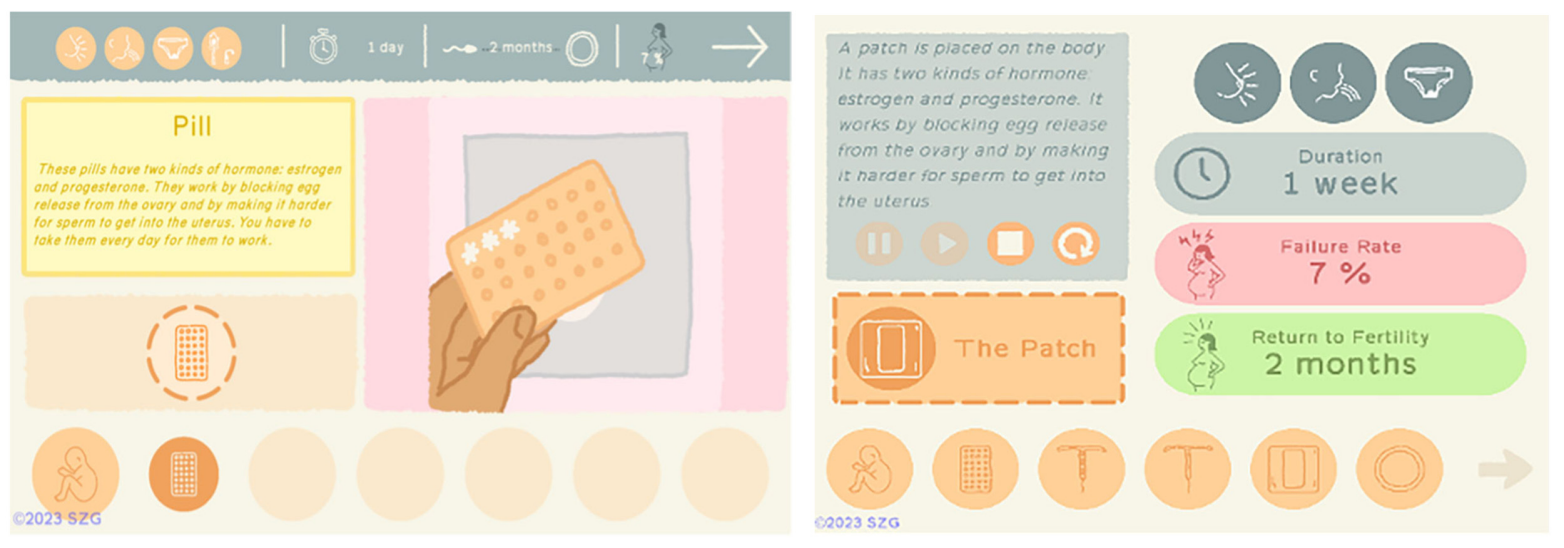
Screenshots from tested and revised Methods sections. Note icons on top of screen at left. Reproduced with permission from SolitonZ Games.

We noted that when participants tested the revised Methods section, they did not pay attention to the icons on the top of the screen that indicate duration, failure rate, and return to fertility for each method. This was also confirmed by participants following playtests. They found the design to be visually overwhelming and could not interpret the icons or pay attention to the information they conveyed. This information led to a redesign of the Methods interface ( Figure 8). In the revision, after the animation demonstrates how a method works, it is replaced by new icons and the information is reinforced by color and placement.

The Couples section of the game will be supplemented given its popularity with participants. The narratives and avatars will be representative of Barbadians with input from our local partners. The parenting groups that include male partners will be more fully characterized giving the men a more active role in the discussion of the methods and how they fit into family life. Increasing the complexity of the conversations in the Couples section will also create an opportunity to provide more detailed information about positive and negative side effects and the likelihood of experiencing them.

### Questionnaire

Given the limited utility of the pre/post questionnaire for assessing knowledge gain, we plan to change both the process and the measure for the RCT. There will no longer be a pre- or post-test quiz. Our primary outcome will instead be a measure of Contraceptive Self-Efficacy (CSE) based on a validated survey published in 2020. The purpose of the RCT is to measure the impact of the WMM game on participants’ sense of agency, education, and empowerment around contraceptive decision-making. For this reason, it is important that the primary outcome not be based on the ultimate choice of contraception, but rather on a measure of personal capabilities around behavior change. CSE is a person’s belief in their own ability to succeed in contraceptive management, initiation, and continued use ( Lillian Whiting-Collins *et al.*, 2020).

The scale to measure Contraceptive Self-Efficacy among women in sub-Saharan Africa (CSESSA) is a modification of the original CSE and is divided into three sub-scales measuring “Husband/partner communication,” “Provider communication,” and “Choosing and managing a method.” This tool was selected for its high relevance to our study and for its focus on participant-directed education and decision-making.

### Limitations

As in a previous deployment, our post-intervention assessment was hampered by some participants choosing not to complete the post-game survey. Providing participants with some form of compensation appears to be necessary to ensure a higher completion rate. We will plan the RCT with some incentives to achieve this. We also acknowledge that having the participants in this deployment having to toggle between two different games may have affected the results. The final version of the game will integrate all sections into one app. All of the participants in this pre-implementation study were better informed about contraception than the actual target audience of game. Motivation to complete the game and interest in the subject matter may differ in the general population.

## Conclusions

The health of childbearing persons is significantly impacted by their ability to make choices about their reproductive health. Thoughtful and informative family planning education can support sexual and reproductive empowerment and self-efficacy. In the era of the internet and digital technology, there are nearly endless options for people to obtain information about contraceptive methods. Healthcare providers who provide contraceptive counseling need to provide detailed information about methods and their side effects as well as counter misinformation that is spread through multiple channels. This study sought to determine if the WMM game is an accepted and effective tool for supporting contraceptive counseling. In order for innovative digital tools to be adopted by heathcare systems and providers, it is important to incorporate their input and needs into the design process and demonstrate that the tool will support and improve the care they provide. The feedback that we collected demonstrates that the WMM game is well-accepted by current and future healthcare providers in Barbados and delivers clearly presented and thorough information in an interactive format. It also helped us identify the parts of the game where information was confusing-- such as the distinction between the copper and hormonal IUDs, and certain side effects – which we addressed in the subsequent revision of the game. Participants in this pilot test were enthusiastic about the game and almost universally desired additional time for gameplay. Several usability flaws and user interface problems were identified and will be corrected. The deployment did not encounter any technical difficulties which suggests that deployment to actual healthcare settings is feasible. The pre/post knowledge gain assessment tool we tested was confusing for participants and did not provide useful data. Qualitative responses provided useful suggestions for improvement and validation of the game as an intervention. This pre-implementation study informed important changes to the deployment process, the WMM game, and the selection of primary outcomes for the RCT.

## Data Availability

OSF: What's My Method Barbados Pilot. https://doi.org/10.17605/OSF.IO/Q2W6U ( Bertozzi & Bertozzi-Villa, 2024). This project contains the following underlying data:
WMMBarbados_Results_Publish.xlsx WMM_Feedback_Publish.xlsx Documentation of the observation This project contains the following extended data:
Pretest Questionnaire- What’s My Method_Contraceptive Knowledge.pdf Posttest Questionnaire – What’s My Method_Player Feedback.pdg Interview and discussion topic guides Data are available under the terms of the Creative Commons Zero "No rights reserved" data waiver (CC0 1.0 Public domain dedication).

## Notes

^1^ https://gcgh.grandchallenges.org/grant/childbearing-intentions-and-family-planning-game

^2^ https://familyplanninggame.qu.edu/index.html
