# Supplementary material for: Supporting contraceptive self-care and reproductive empowerment with a digital health game in Barbados: Development and Pre-implementation study for What’s My Method?
Source: Gates Open Res. 2024 Nov 21;8:47. Originally published 2024 May 28. [Version 2] doi: 10.12688/gatesopenres.15376.2 (PMC11772015; doi:10.12688/gatesopenres.15376.2)
Supplement: Peer review history

# Reviewer report: Reviewer response for version 2

**DOI:** 10.21956/gatesopenres.17693.r38503

**Published:** 2024-12-20

**Authors:** Chi-Chi Undie

**Affiliations:** 1 Population Council, Nairobi, Kenya

I’m fine with moving ahead to approve this paper for indexing. I have no further comments.

Is the rationale for developing the new method (or application) clearly explained?

Partly

Is the description of the method technically sound?

Partly

Are the conclusions about the method and its performance adequately supported by the findings presented in the article?

Partly

If any results are presented, are all the source data underlying the results available to ensure full reproducibility?

Partly

Are sufficient details provided to allow replication of the method development and its use by others?

Partly

Reviewer Expertise:

NA

I confirm that I have read this submission and believe that I have an appropriate level of expertise to confirm that it is of an acceptable scientific standard.

# Reviewer report: Reviewer response for version 2

**DOI:** 10.21956/gatesopenres.17693.r38504

**Published:** 2024-12-19

**Authors:** Betzabe Torres-Cortés

**Affiliations:** 1 Psychology, Universidad de O'Higgins (Ringgold ID: 519092), Rancagua, O'Higgins Region, Chile

I have no further comments

Is the rationale for developing the new method (or application) clearly explained?

Yes

Is the description of the method technically sound?

Partly

Are the conclusions about the method and its performance adequately supported by the findings presented in the article?

Yes

If any results are presented, are all the source data underlying the results available to ensure full reproducibility?

No

Are sufficient details provided to allow replication of the method development and its use by others?

No

Reviewer Expertise:

Designing Health Interventions from an Implementation Science Perspective

I confirm that I have read this submission and believe that I have an appropriate level of expertise to confirm that it is of an acceptable scientific standard.

# Reviewer report: Reviewer response for version 2

**DOI:** 10.21956/gatesopenres.17693.r38505

**Published:** 2024-11-23

**Authors:** Summer Mengelkoch

**Affiliations:** 1 University of California, LA, CA, USA

Great job implementing feedback!

Is the rationale for developing the new method (or application) clearly explained?

Yes

Is the description of the method technically sound?

Yes

Are the conclusions about the method and its performance adequately supported by the findings presented in the article?

Partly

If any results are presented, are all the source data underlying the results available to ensure full reproducibility?

Yes

Are sufficient details provided to allow replication of the method development and its use by others?

Partly

Reviewer Expertise:

I am a social psychologist, with extensive experience in women's health research, including impacts of hormones and hormonal contraceptive use on stress reactivity, persistence, and depression. I also have extensive experience in psychoneuroimmunology and implementation of clinical interventions.

I confirm that I have read this submission and believe that I have an appropriate level of expertise to confirm that it is of an acceptable scientific standard.

# Reviewer report: Reviewer response for version 1

**DOI:** 10.21956/gatesopenres.16726.r37843

**Published:** 2024-09-25

**Authors:** Betzabe Torres-Cortés

**Affiliations:** 1 Psychology, Universidad de O'Higgins (Ringgold ID: 519092), Rancagua, O'Higgins Region, Chile

Summary: The study presents the design and preliminary evaluation of a gaming intervention for reproductive empowerment and contraceptive self-efficacy.

 Title: it should not include the word "development," as the manuscript does not describe the design of the intervention (which was completed prior to the study)

 Methods:

 - I understand that the theoretical foundations refer to what was used in designing the intervention. However, I suggest moving them to the Introduction section, as the Methods section should only include aspects directly related to the study, not the design of the intervention itself.

 - A clearer definition of the methodology is needed. Is it a mixed-methods study or a study with both qualitative and quantitative phases? I recommend referring to established authors for guidance.

 - Design and pre-implementation studies can indeed include a sample. I recommend reviewing this statement.

 - I recommend including a specific section for data collection techniques, as they are currently integrated into the "Procedure" section, which is not appropriate

 - Observation and documentation of participant behavior are data collection techniques. It is recommended to include them as such and provide a detailed description: Was a structured guideline used for observation and documentation, or was it done in an unstructured manner?

 - The description of the pre-test should be included in the Data Collection Techniques section, not in the Analysis section

 - The Analysis section needs to be revised, as it currently does not specify the techniques used for analyzing quantitative and qualitative data. It is unclear how the results were obtained.

 Results:

 - I recommend highlighting which results correspond to each data collection technique (focus groups, observation, documentation and pre-post test). The current presentation requires the reader to infer this information.

 Methods and results:

 - The study indicates that it is based on the EPIS framework; however, the Methods/Results section does not reference the stages of this framework or specify which parts of the study correspond to each stage. I suggest including this information.

 Overall, it can be said that the study lacks replicability: its methodology is unclear, it claims to be based on the EPIS framework but does not incorporate this framework in the methods or results sections, it does not adequately identify its data collection techniques, and it lacks detailed descriptions of its data analysis methods. As a result, it is impossible to determine the source of the results or how to replicate the study.

Is the rationale for developing the new method (or application) clearly explained?

Yes

Is the description of the method technically sound?

Partly

Are the conclusions about the method and its performance adequately supported by the findings presented in the article?

Yes

If any results are presented, are all the source data underlying the results available to ensure full reproducibility?

No

Are sufficient details provided to allow replication of the method development and its use by others?

No

Reviewer Expertise:

Designing Health Interventions from an Implementation Science Perspective

I confirm that I have read this submission and believe that I have an appropriate level of expertise to state that I do not consider it to be of an acceptable scientific standard, for reasons outlined above.

# Response

**Published:** 2024-10-15

**Authors:** Elena Bertozzi

**Affiliations:** Game Design & Development, Quinnipiac University, Hamden, CT, USA

Thank you very much for this helpful review that informed us about gaps in the way that we explained the process and purpose of the study. We have made significant edits to the text to clarify and address your notes in detail below.

  

1. “Development”

Game design is an iterative process wherein the game is repeatedly tested and modified in response to feedback from the intended audience. The game intervention in this study was not completed prior to the study. The study was an important step in the process of ensuring that the game meets the needs of the healthcare professionals who would be deploying it within a health care setting, that it effectively communicates the desired information, and that the intended audience is motivated to engage and interact with it. The latter half of the paper details the changes that we made to the intervention as a result of this work. Thank you for pointing out that we did not make this clear in the abstract and introduction. We have modified the text to clarify. Given your expertise in Implementation Science, your comments are much appreciated.

  

1. Methods

The theoretical foundations we cite do inform the study. The study is part of the process of improving the WMM game itself and assessing stakeholder feedback on it.

 We included more citations and details about the methods of the study.

 The analysis section was revised to provide the requested information

  

1. Results

Tables and captions were added to clarify source and type of data and analysis

 The note that we should include more of the EPIS framework in the paper is also very helpful. We have added information about the process steps of the framework and how implementation science differs from other kinds of study design.

 Given that the study aims to “conduct a formative evaluation of the What’s My Method? (WMM) game intervention as a tool to support contraceptive counseling” as we state in the abstract, the data that we collected was focused on assessing the value of our proposed questionnaire as a tool for quantifying knowledge gain and on the attitudes of the healthcare providers and intended audience for this specific game. Thus the only numerical data we are reporting are 1) A comparison of the pre and post test scores for knowledge gain, and the means of the Likert scale scores for acceptability of the intervention. Based on your and the other reviewers’ feedback we have edited those tables for clarity.

 In terms of replicability – as a development and pre-implementation study of a very specific game intervention in a specific healthcare environment, this study is not designed to be replicable. It is a step in the process of preparing for the RCT of the intervention which will be. Although many games for health have been designed and developed, few have actually been integrated into healthcare environments. We hope that this paper will help support others who seek to do so.

 Thanks, we appreciate your comments and expertise.

# Reviewer report: Reviewer response for version 1

**DOI:** 10.21956/gatesopenres.16726.r37838

**Published:** 2024-09-17

**Authors:** Chi-Chi Undie

**Affiliations:** 1 Population Council, Nairobi, Kenya

This paper presents developmental and pre-implementation evidence to inform an upcoming RCT around the use of an exciting game intervention to enhance contraceptive self-efficacy in Barbados. It is an interesting paper which could be strengthened by outlining the methods more clearly. Suggestions for rectifying this and other issues are provided below.

- The rationale for developing the game intervention is convincingly laid out in the Background section, with extensive arguments building up to the need for new studies that demonstrate self-efficacy related to other contraceptive methods (beyond condoms alone), which in turn result in increased use of those methods. In addition, evidence around lower than desired CPR in the study context, the use of abortion as birth control, etc., is outlined. These arguments imply that the game intervention will play a key role in the uptake of contraception. However, in the Questionnaire section of the paper, the authors indicate that “[I]t is important that the primary outcome not be based on the ultimate choice of contraception, but rather on a measure of personal capabilities around behavior change.” This assertion is fine in its own right, but it is misaligned with the extensive arguments laid out in the Background section. Some of the language in Background section therefore needs to be tempered to maintain the point that the end goal has to do with personal capabilities around behavior change, rather than actual contraceptive use/uptake/choice.
- The Analysis section does not provide a description of the qualitative data analysis. The procedures for conducting the focus group discussions (FGDs) are also not laid out. They are also referred to as an ‘informal discussion’ in the Procedures section, which makes it unclear to the reader whether several FGDs were carried out, or a single informal discussion. The authors do not indicate how many FGDs were conducted, and with which respondent categories. Little information is provided on how exactly the observations were carried out (were they structured in any way?). In general, the procedures, analysis, and results around the qualitative data seem to have been almost overlooked/omitted, compared to what obtains for the quantitative data. It makes the conclusions less persuasive than they could have been. The vagueness around the qualitative methods makes them challenging for others to use, replicate, and reproduce.
- Participants section: The first sentence indicates that ‘no sampling was involved.’ Instead, it should indicate that a combination of convenience sampling and purposive (or purposeful) sampling was employed – convenience because whoever volunteered was enrolled in whatever numbers, but purposeful because the volunteers did have to represent specific categories of people in order to be eligible to participate.
- The Conclusions indicate that ‘the game is well-accepted by the target audience and delivers clearly presented and thorough information in an interactive format.’  This seems to gloss over the confusion participants experienced in distinguishing between the copper and hormonal IUDs, and in understanding some of the contraceptive self-efficacy parameters, for example. It would be best to temper the language in this sentence. Furthermore, the target audience of childbearing people used by the study is arguably a really select group. The study used youth contraception ambassadors and nursing students, rather than just ‘regular’ youth, who are probably not as sensitized as the ambassadors or nursing students, and who are, therefore, really the ones that need to develop contraceptive self-efficacy.  The use of these groups, as opposed to less informed youth should be discussed in the Limitations section.
- Male participants wanted to see a more inclusive approach to men featured in the game stories. What does this mean for the reproductive autonomy of childbearing persons? Are there any implications? If so, it would be helpful to reflect on this in the Discussion.
- Design section: This section currently states: ‘We conducted focus group discussions and collected both qualitative and quantitative data.’ Since FGDs are qualitative data, it should instead state something like: ‘We conducted both qualitative (focus group discussions) and quantitative (pre- and post-test survey) data.’
- “If the selection is correct.” This sentence is hanging, and is probably meant to be a part of the sentence that precedes it.

Is the rationale for developing the new method (or application) clearly explained?

Partly

Is the description of the method technically sound?

Partly

Are the conclusions about the method and its performance adequately supported by the findings presented in the article?

Partly

If any results are presented, are all the source data underlying the results available to ensure full reproducibility?

Partly

Are sufficient details provided to allow replication of the method development and its use by others?

Partly

Reviewer Expertise:

NA

I confirm that I have read this submission and believe that I have an appropriate level of expertise to confirm that it is of an acceptable scientific standard, however I have significant reservations, as outlined above.

# Response

**Published:** 2024-10-15

**Authors:** Elena Bertozzi

**Affiliations:** Game Design & Development, Quinnipiac University, Hamden, CT, USA

We very much appreciate the note that we needed to clarify how user feedback was collected. This should also be clearer now that we have included more details about the EPIS framework as suggested by Reviewer #3. We list the revisions below in the order suggested by your review.

- Modified the first paragraph of the Methods section to clarify that we did not conduct formal focus group sessions where qualitative data was collected. The qualitative responses we report all come from the questionnaires.
- Thank you very much for this helpful language re: sampling which we included.
- Added text in the Conclusion explaining how we modified the game in response to player confusion in the specified areas and how this fits into the EPIS framework.
- Added a qualification to the Limitations Section and also noted it in the Participants section.
- We edited the section about including males to reflect the diversity in parenting arrangements.
- We reworded the Design section to clarify this point and appreciate your catching the typo.

Thank you very much for your time and attention to our work. We appreciate your help in improving the paper.

# Reviewer report: Reviewer response for version 1

**DOI:** 10.21956/gatesopenres.16726.r37423

**Published:** 2024-08-05

**Authors:** Summer Mengelkoch

**Affiliations:** 1 University of California, LA, CA, USA

The authors present the results of a pre-implementation study assessing What’s My Method (WMM), a digital health game designed to teach users about contraception and enhance reproductive self-efficacy. After describing the game, the authors discuss primarily qualitative outcomes assessed in pre-implementation users ( *N* = 42), and some non-significant outcomes of contraceptive knowledge tests. The game seems quite useful and interesting, and certainly deserves additional study. I was impressed with the design and approach here. In general, the game and study are well-founded and I am excited to hear the results of the RCT. Although of acceptable standards, there are a few points which I believe require additional clarification and reorganization before this is suitable for indexing. Overall, I found this to be a well written report and an interesting, if preliminary, research study. Below, I detail my major comments and suggestions and hope that my feedback improves the overall quality of the current report.

 Major Comments (in order of importance):

 Discussion:

1. The knowledge gleaned from pre/post quizzes is really important to research. Even if the results don't come out as expected or desired, that is no reason to pivot away and leave knowledge entirely unassessed. Instead, I would HIGHLY recommend also including a pre/post test of knowledge, but considering it a secondary (or even exploratory) outcome for the RCT. Not including it at all because the results came out weird in a small and unincentivized group of participants seems misguided. I think the authors will miss out on really valuable information by entirely relying on efficacy measures. Accurate knowledge is far more empowering than a perception of knowledge that leaves users unprepared to make informed contraceptive choices. 

Results:

1. Table 2 is very difficult to understand. The authors would benefit from considering and including information that will be useful to the reader. First, the authors should label variable 1 and variable 2 as pre/post scores, if that is what they are. I’m assuming that the mean is the mean of the number correct out of five? So higher scores here are better? The authors should be clear about the numbers they are presenting and how they are scored. It is standard to round off values at two decimal places. What measure of variance is listed? Why did the authors hypothesize a mean difference of 0? It seems that the statement might represent a null hypothesis, which is typically not the focus in a paper of this nature. Why are there more t-values than p-values? Perhaps the last line is missing? Instead of presenting all possible t-statistics, the authors should be presenting the stat that is the most appropriate for their analysis (likely the two-tailed) or explain to the reader why multiple stats are presented.
2. Instead of only presenting values for a few of the feedback questions, all should be presented in a table, as these are the key results interpreted here.

Introduction:

1. At the outset of the paper, the authors explain that contraceptive counseling and education should be implemented through a “lens of reproductive empowerment”. They follow with some of these statements: “Ideally, this counseling also reviews the many non-contraceptive benefits of hormonal birth control methods,” … and “Childbearing women receive advice from older women propagating unsupported concerns about side effects, which makes them reluctant to adopt modern methods.” without citations, implying these are the authors’ observations or opinions. It is not empowering to highlight benefits without discussing risks, nor to dismiss very real and well-supported side effects of hormonal contraceptives as “unsupported concerns” because they don’t occur in all women under all conditions. It is far more empowering to women to explain the balance of potential costs alongside potential benefits of contraceptive options, allowing women to make fully informed choices that align with their priorities and values. The authors should consider the tone at the outset of the paper, and soften or modify these statements, as the purpose of this paper is not to glorify hormonal contraceptives, but rather to promote reproductive empowerment through gamified contraceptive education in a population where these actions are likely to be very beneficial. 
2. Before explaining the history and development of the WMM game, it would be helpful to give the reader a brief overview of the game, either just before or just after the aims.
3. Figure 1: Update the figure to be accurate... sperm aren't produced in fallopian tubes, as the figure implies (I assume this is just a labeling error and not a real screenshot from the game)
4. Figure 4: Please make the text legible, even if it requires editing the screengrab by overlaying text that is less blurry!

Methods:

1. The theoretical frameworks section belongs in the introduction, not the methods.
2. Participants: the statement that no sampling was involved is a bit overstated. It was not random sampling, but sampling still occurred.

Minor comments (the authors should not feel obligated respond to these minor comments unless they would like to, they are simply provided in case they are helpful in improving the quality of the manuscript):

 Abstract:

1. “Results indicate that the WMM game is well-reviewed and accepted” Did the authors mean well-received?
2. Last line: instead of referencing “the” upcoming RCT, “an” upcoming RCT is more appropriate.

Introduction:

1. First line: typical to spell out abbreviations the first time they are used (WHO)
2. “Implementing contraceptive counseling and education through the lens of reproductive empowerment requires that the focus of the intervention be on the childbearing persons and their needs.” Remove the “the” before intervention
3. If this was meant to be a header, please format as such “Demonstrating the impact of interventions on reproductive empowerment is complicated.”
4. The first time the WMM acronym is used, please define it so the reader knows what it is!
5. “Game development is an iterative process requiring testing on the target audience during the production cycle to ensure that the game is fun, easy to use and understand, and achieves the study goals.” Here, I don’t think “achieves the study goals” is a part of game development. Perhaps the word game goals would be more accurate.

Methods:

1. Consider using a screen grab for Fig 6 in which you have removed the blue lines indicating a grammatical error in #1
2. The statement: “2 participants did not complete the pre-test and 7 did not complete the post-test. Thus our comparison of pre/post results utilized a data set of 33 responses.” Does not belong under the header “ethical considerations”

Discussion: really impressed with the way user feedback was used to improve the game!

Is the rationale for developing the new method (or application) clearly explained?

Yes

Is the description of the method technically sound?

Yes

Are the conclusions about the method and its performance adequately supported by the findings presented in the article?

Partly

If any results are presented, are all the source data underlying the results available to ensure full reproducibility?

Yes

Are sufficient details provided to allow replication of the method development and its use by others?

Partly

Reviewer Expertise:

I am a social psychologist, with extensive experience in women's health research, including impacts of hormones and hormonal contraceptive use on stress reactivity, persistence, and depression. I also have extensive experience in psychoneuroimmunology and implementation of clinical interventions.

I confirm that I have read this submission and believe that I have an appropriate level of expertise to confirm that it is of an acceptable scientific standard, however I have significant reservations, as outlined above.

# Response

**Published:** 2024-10-15

**Authors:** Elena Bertozzi

**Affiliations:** Game Design & Development, Quinnipiac University, Hamden, CT, USA

Thank you for this thorough and thoughtful feedback. We have implemented your suggestions. Please see details below.

 Suggestion for finding a way to effectively measure knowledge gain noted.

 Results Section

1. Reformatted Table 2 to improve legibility and include requested data
2. Added Table 4 with values for all feedback questions.

Introduction:

1. Added text about risks and citation for benefits of hormonal birth control. We revised text to make clear that these are non-scientific concerns about contraceptive side effects.
2. Added a brief description of WMM before the Aims section
3. Corrected screenshot label – Thanks for catching this!
4. Redid screenshots and improved resolution. (see figures Figure 1B, and new Figures 4a and 4b)

Methods:

1. Moved theoretical frameworks section to appropriate location
2. Eliminated phrase stating “no sampling” and revised text

Thank you for the individual editing suggestions all of which we implemented. We very much appreciate your time and assistance in improving our paper.
